# Green Tea Polyphenol (−)-Epigallocatechin Gallate (EGCG) Attenuates Neuroinflammation in Palmitic Acid-Stimulated BV-2 Microglia and High-Fat Diet-Induced Obese Mice

**DOI:** 10.3390/ijms20205081

**Published:** 2019-10-13

**Authors:** Limin Mao, Danielle Hochstetter, Liyun Yao, Yueling Zhao, Jihong Zhou, Yuefei Wang, Ping Xu

**Affiliations:** Tea Research Institute, Zhejiang University, Hangzhou 310058, China; maolm@zjtea.com (L.M.); 21816158@zju.edu.cn (D.H.); 21716065@zju.edu.cn (L.Y.); 11616050@zju.edu.cn (Y.Z.); zhoujihong@zju.edu.cn (J.Z.)

**Keywords:** tea polyphenols, saturated fatty acids, obesity, hypothalamus, inflammation

## Abstract

Obesity is closely associated with neuroinflammation in the hypothalamus, which is characterized by over-activated microglia and excessive production of pro-inflammatory cytokines. The present study was aimed at elucidating the effects of (−)-epigallocatechin gallate (EGCG) on palmitic acid-stimulated BV-2 microglia and high-fat-diet-induced obese mice. The results indicated the suppressive effect of EGCG on lipid accumulation, pro-inflammatory cytokines (TNF-α, IL-6, and IL-1β) release, and microglial activation in both cellular and high-fat-diet rodent models. These results were associated with lower phosphorylated levels of the janus kinase 2/signal transducers and activators of transcription 3 (JAK2/STAT3) signaling pathway. In conclusion, EGCG can attenuate high-fat-induced hypothalamic inflammation via inhibiting the JAK2/STAT3 signaling pathways in microglia.

## 1. Introduction

The prevalence of obesity is dramatically increasing worldwide in recent decades [1]. The consumption of high-fat diet (HFD) is a causative factor for being overweight or obese [2]. HFD is rich in saturated fatty acids (SFA), such as palmitic acid (PA), stearic acid, and lauric acid. An excess amount of SFA in the diet is a key pathogenic link between HFD-induced obesity and concomitant chronic low-grade systemic inflammation [3].

The hypothalamus section of the central nervous system (CNS) regulates lipid homeostasis and energy metabolism [4,5,6]. Several hypothalamic nuclei, namely the arcuate nucleus (ARC) and the paraventricular nucleus (PVN), are involved in maintaining energy balance [7]. Microglia are the major macrophages in the hypothalamus, which can initiate rapid morphological and functional transformations in response to excessive dietary SFA, triggering the abundant release of inflammatory cytokines, followed by a series of inflammatory reactions [8]. Lately, anti-inflammatory therapies based on the microglia activation process have shown high prospects for the treatment of obesity and metabolic syndromes [6].

Green tea, a widely consumed beverage, contains many bioactive constituents, among which (−)-epigallocatechin gallate (EGCG) is one of the most potent compounds with anti-obesity effects [9,10]. Many studies have reported that EGCG increases energy expenditure, alleviates adipose insulin resistance, induces adipocyte apoptosis, and inhibits preadipocyte differentiation [11]. Nevertheless, a detailed central modulation mechanism of EGCG on HFD-induced neuroinflammation has not yet been elucidated.

In the present study, the influence of EGCG was evaluated on SFA-mediated lipid accumulation and microglia-mediated hypothalamic inflammation in PA-stimulated BV-2 cells and HFD-induced obese mice. Our results show that EGCG attenuates HFD-induced neuroinflammation via suppressing the JAK2/STAT3 signaling pathway. This outcome would provid insight into the neurological mechanism of the anti-obesity activity of EGCG.

## 2. Results

### 2.1. EGCG Attenuates Lipid Accumulation and Inflammatory Responses in PA-Stimulated BV-2 Cells

**Figure 1:**
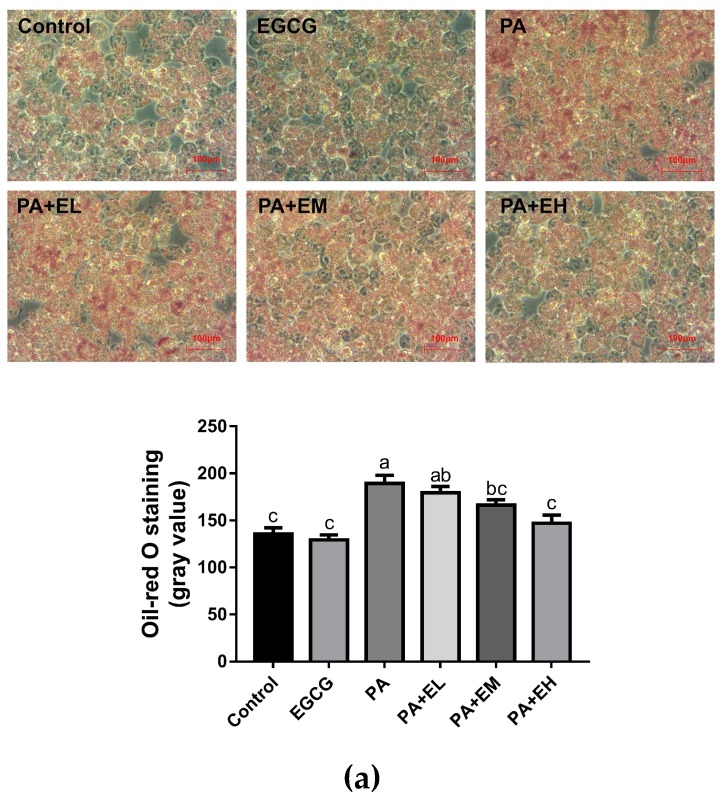

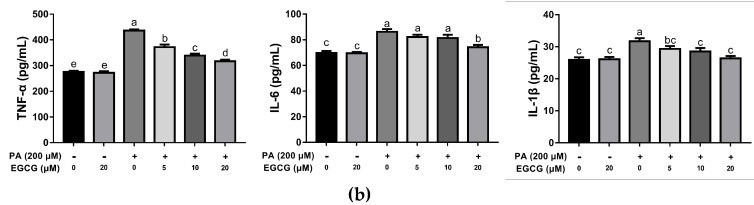
Effects of (−)-epigallocatechin gallate (EGCG) on lipid accumulation and inflammatory responses of PA-stimulated BV-2 cells. BV2 cells were pretreated with different dosages of EGCG (EL: low concentration of EGCG (5 μM), EM: medium concentration of EGCG (10 μM), EH: high concentration of EGCG (20 μM)) for 2 h and then stimulated with 200 μM palmitic acid (PA) for 24 h. (**a**) Lipid distribution in BV-2 cells was observed by oil red O staining and analyzed with ImageJ; lipids were stained red; (**b**) Levels of TNF-α, IL-6, and IL-1β in the conditioned medium were measured by ELISA. Data are means ± SEM of three independent experiments performed in triplicate. Different superscript letters indicate significantly different means at *p* < 0.05 (a > b > c > d).

PA induces lipid accumulation and pro-inflammatory cytokine expression in microglia [12]. We first investigated whether EGCG could reduce lipid accumulation in PA-stimulated BV-2 cells with oil red O staining. The tested concentrations of PA and EGCG in all the experiments were subjected to the MTT (3-(4,5)-dimethylthiahiazo (-z-y1)-3,5-di- phenytetrazoliumromide) assay, and EGCG at concentrations ranging from 5 to 20 μM did not affect the cell viability (data not shown). According to Figure 1a, oil red O staining revealed that the accumulation of lipid droplets in BV-2 cells was increased substantially after PA treatment, which is clearly improved in groups with EGCG pretreatment. In addition, ELISA showed lower levels of TNF-α, IL-6, and IL-1β in the EGCG pretreated groups than the PA group, and with the increase of EGCG concentration, the activity increased (Figure 1b). These data suggest that EGCG suppressed the PA-induced excessive lipid accumulation and inflammatory response in microglia in a dose-dependent manner. In addition, the concentration gradient assay suggested that all three doses of EGCG could attenuate the PA-induced excessive lipid accumulation and inflammatory responses, while the highest dose produced the most effective inhibition.

### 2.2. EGCG Suppresses Phosphorylation of JAK2 and STAT3 in PA-Stimulated BV-2 Cells

**Figure 2:**
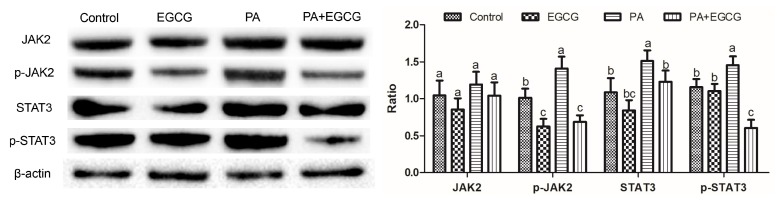
Inhibition effect of (−)-epigallocatechin gallate (EGCG) on JAK2/STAT3 signaling activation in PA-stimulated BV-2 cells. BV-2 cells were pretreated with 20 μM EGCG for 2 h and then stimulated with 200 μM palmitic acid for 24 h. Representative Western blots for JAK2 and STAT3 phosphorylation in BV-2 cells were shown. Data are means ± SEM of three independent experiments performed in triplicate. Different superscript letters indicate significantly different means at *p* < 0.05 (a > b > c).

Phosphorylation of STAT3 can be induced by JAK2, and inactivation of STAT3 triggers an inflammatory response [13,14]. The levels of related proteins were evaluated for investigating whether EGCG inhibited the neuroinflammation via the JAK2/STAT3 pathway. The western blot analysis (Figure 2) demonstrated that the phosphorylation of JAK2 and STAT3 were significantly upregulated by PA compared to the control group, whereas EGCG prevented the phosphorylation of JAK2 and STAT3 in PA-stimulated BV-2 cells. These data establish the JAK2/STAT3 pathway as an effective target for EGCG in PA-stimulated BV-2 cells.

### 2.3. EGCG Ameliorates HFD Induced Obesity

**Figure 3:**
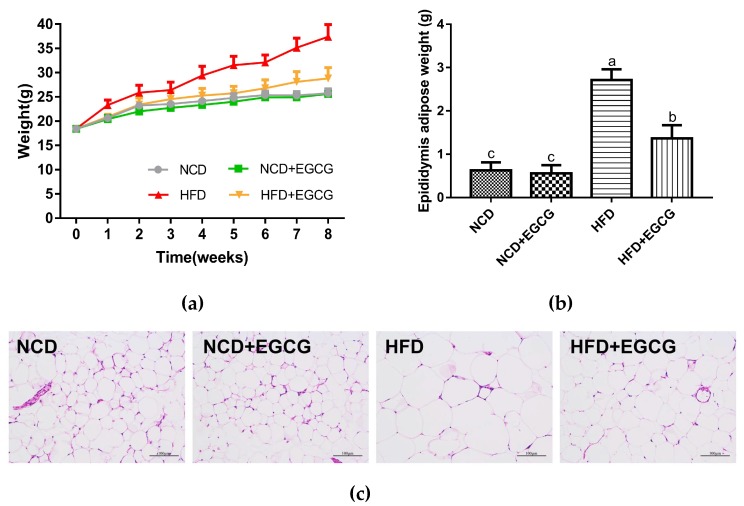
Effect of (−)-epigallocatechin gallate (EGCG) on HFD-induced obesity. (**a**) Weight variation tendency of different groups; (**b**) Weight of epididymis adipose; (**c**) Representative hematoxylin and eosin (H&E) staining of epididymis adipose sections. Data are means ± SEM (*n* = 6). Different superscript letters indicate significantly different means at *p* < 0.05 (a > b > c).

In the present study, obesity in male C57BL/6J mice was induced by HFD during an 8-week period. EGCG-treated (fed with a 60 kcal% high-fat diet supplemented with 1% EGCG) mice showed an obvious reduction in body weight, lipid deposition, and epididymal adipocytes size (Figure 3a–c).

Serum glucose and lipid levels were also measured. Compared to HFD mice, EGCG treated group significantly restored the blood glucose, total cholesterol (TC), and triglyceride (TG) levels under fasting conditions (Table 1). These results illustrate the anti-obesity potential of EGCG.

### 2.4. EGCG Alleviates the Obesity-Associated Neuroinflammation of the Hypothalamus

**Figure 4:**
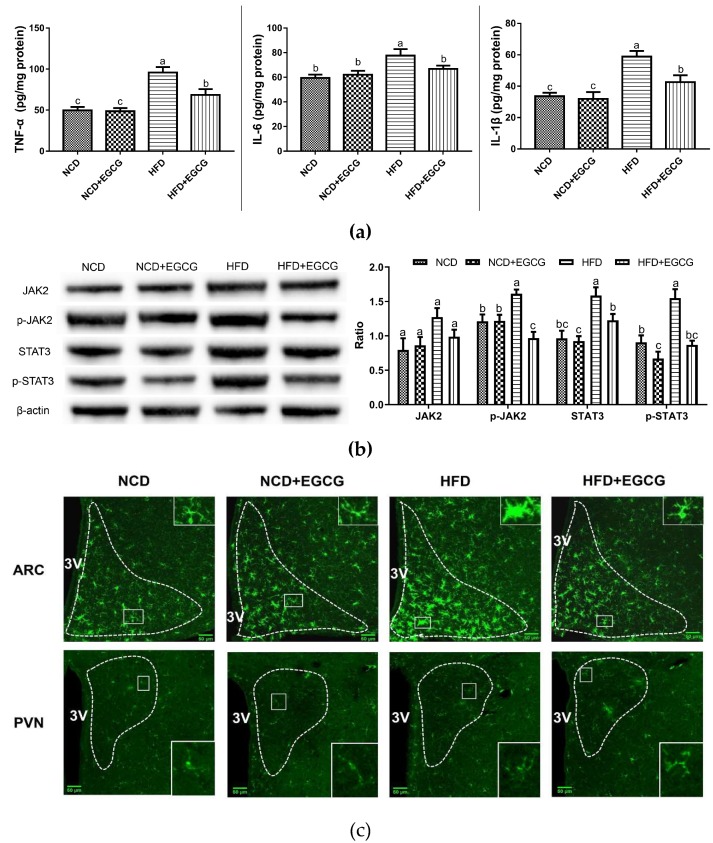
Inhibition effect of (−)-epigallocatechin gallate (EGCG) on obesity-associated neuroinflammation of hypothalamus. (**a**) Concentrations of TNF-α, IL-6, IL-1β in the hypothalamus; (**b**) Representative Western blots for JAK2 and STAT3 phosphorylation levels in the hypothalamus; (**c**) Representative micrographs of immunofluorescence labeling for Iba1 in the hypothalamic arcuate nucleus (ARC) and paraventricular nucleus (PVN) (outlined by white dashed lines) and higher magnification insets (outlined by white solid lines). Data are means ± SEM of three independent experiments performed in triplicate (*n* = 3). Different superscript letters indicate significantly different means at *p* < 0.05 (a > b > c).

The hypothalamus centrally controls the energy homeostasis. Recent studies have revealed the involvement of chronic low-grade hypothalamic inflammation in the modulation of DIO (diet-induced obesity) [15,16]. In our study, the EGCG-treated mice presented a marked decline of the inflammatory cytokine levels (TNF-α, IL-6, and IL-1β) in the hypothalamus than the HFD group (Figure 4a,b). The molecular underlying mechanisms related to these changes were elucidated through western blot analysis, which measured the expression of the key factors in the JAK2/STAT3 pathway. Figure 4b shows that the phosphorylation of JAK2 and STAT3 was noticeably upregulated by HFD, which could be suppressed by EGCG. Thus, EGCG can alleviate the obesity-associated neuroinflammation of the hypothalamus via regulating JAK2/STAT3 signaling pathway.

Microglia activation is associated with the development of obesity-induced hypothalamic inflammation [17,18]. EGCG induced inhibition of microglial activation in hypothalamic ARC and PVN was identified by evaluating Iba-1 (a microglial marker) immunofluorescence labeling. Previous studies have identified two major morphological types of microglia, namely “ramified resting” and “activated amoeboid”, which are associated with the proinflammatory, cytotoxic, and immunoregulatory functions [19]. Ramified microglia with numerous thin, long processes are known to transit into rounded soma with little or no branching when stimulated under pathological conditions [20]. Figure 4c shows a significant increase in Iba-1 labeling with amoeboid morphological transitions in the hypothalamic ARC of HFD mice. Conversely, many quiescent microglial cells with small cell bodies and ramified processes were observed in the hypothalamic ARC of control and EGCG-treated mice (Figure 4c). In addition, no significant difference in microglial activation was observed in PVN (Figure 4c).

To sum up, dietary supplementation with EGCG effectively alleviated obesity-associated hypothalamic inflammation mediated via down-regulation of the JAK2/STAT3 signaling pathway, which is most likely related to the microglial inflammatory signaling in hypothalamic ARC instead of PVN.

## 3. Discussion

Many studies support that tea polyphenols play as an effective role in lowering the risk of obesity, diabetes, and metabolic syndrome. Several mechanisms have been proposed, such as reducing adipocyte proliferation, impeding the absorption of fat, and promoting the oxidation of fatty acids [21,22,23,24]. Our previous studies have shown that EGCG ameliorated HFD-induced obesity without affecting the food and energy intake, suggesting that EGCG might play an important role on promoting energy expenditure [25]. The central nervous system, particularly the hypothalamus, is the major regulator of energy metabolism. However, the role of EGCG in regulating the central nervous system in HFD-induced obesity with a detailed underlying molecular mechanism remains unclear.

Inflammatory responses and dysfunction in the hypothalamus are the common features of HFD-induced obesity models [26,27,28]. Microglia participates in a cell-mediated hypothalamic immune response to dietary excess; though, the mechanisms by which microglia inflicts neuronal injury and functional disorder should be thoroughly studied [18,29,30]. Palmitic acid is a common SFA in human diets, which is accumulated in the hypothalamus after HFD consumption [12,31]. Based on the previous method [32], PA-stimulated BV-2 cells were utilized for investigating the effect of EGCG on HFD-induced microglial inflammation. EGCG diminished the excessive lipid accumulation and the production of proinflammatory cytokines in this model. These results were also confirmed in the subsequent HFD-induced obese mice model. Also, EGCG inhibited the activation of microglia in the hypothalamic ARC. Consistent with our previous study [25], these data suggest that EGCG ameliorated metabolic disorders via reducing hypothalamic inflammatory responses, and microglial-induced central sensitization in the hypothalamic ARC. Hypothalamic ARC has been regarded as one of the core control centers of metabolism, in which proopiomelanocortin (POMC) and agouti-related neuropeptide (AgRP) neurons respond to signaling cues from the periphery, such as leptin, insulin, or ghrelin [33]. In previous studies, chronic low-grade hypothalamic inflammation was proved to disrupt peripheral insulin and leptin signaling and glucose homeostasis [34,35]. The neurological mechanism of EGCG in controlling obesity may be related to the regulation of these neuropeptides and related hormones.

STAT3 is a representative of signal transducer and activator of transcription (STAT) family, which can be phosphorylated by Janus kinase (JAK) in response to ligand (LPS, PA, etc.) stimulation [36]. Phosphorylation of STAT3 is closely related with inflammation, oxidative stress, and apoptosis [37]. Recent studies have proposed that proinflammatory responses in microglia are controlled by the upstream JAK2/STAT3 pathway, which is involved in the central regulation of hypothalamic inflammation in high-fat-diet-induced obese rodents [14,36,38,39,40,41]. Several studies have reported that plant polyphenols, such as Ougan polymethoxyflavones [40], proanthocyanidins [42], and green tea polyphenol [25], could reduce neuroinflammation through the JAK2/STAT3 pathway. Consistent with previous studies, our results demonstrate that EGCG decreases the abnormally elevated protein expression of p-JAK2 and its downstream factor p-STAT3 induced by the HFD, and then time activated STAT3 translocates into the nucleus for regulating the expression of inflammatory-related target genes [43]. These results indicate that EGCG was involved in the regulation of the JAK2/STAT3 signaling pathway negative feedback, or the activation of negative regulators of the JAK2/STAT3 signaling pathway.

**Figure 5:**
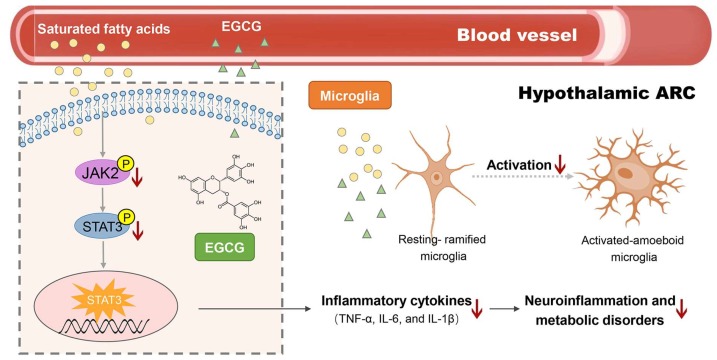
Possible neurological mechanisms of (−)-epigallocatechin gallate (EGCG) on HFD-induce obesity.

In summary, the present study demonstrated that EGCG decreased the HFD-induced neuroinflammation and microglial activation in the hypothalamic ARC region, which may be actualized via altered JAK2/STAT3 signaling in the hypothalamus (Figure 5). These findings elucidate the neurological mechanism of the anti-obesity effect of EGCG, suggesting that EGCG might be a potential therapeutic agent for the treatment of obesity-mediated neuroinflammation. Specific chemical inhibitors for the JAK2/STAT3 pathway could be used in cell models and mice models to confirm further if such changes in cytokine levels and microglial activation levels are dependent on JAK2/STAT3 signaling upon EGCG treatment. Meanwhile, more research is required for elucidating the effects of EGCG on the interaction of microglia, astrocytes, related neuropeptide, and peripheral hormone signaling in hypothalamic inflammation for further clarification of the precise neuronal networks involved in metabolic regulation.

## 4. Materials and Methods

### 4.1. Materials

EGCG (purity > 95%) was obtained from Huzhou Rongkai Foliage Extract Co., Ltd. (Huzhou, China). A fatty acid-albumin complex solution containing palmitic acid (PA, Sigma–Aldrich, St Louis, MO, USA) and fatty acid-free bovine serum albumin (BSA, Sigma, St Louis, MO, USA) was prepared as described previously [44]. All cell culture reagents were purchased from Gibco (Grand Island, NY, USA). Primary antibodies specific for NF-κB, phospho-NF-κB, IκB-α, phospho-IκB-α, Stat3, phosphoStat3, Jak2, and phospho-Jak2 were purchased from Cell Signaling Technology (Beverly, MA, USA), and Iba-1 was obtained from Wako Pure Chemical Industries, Ltd. (Osaka, Japan). D12450J normal chow diet and D12492 high-fat diet were purchased from Research Diets, Inc. Co., Ltd. (New Brunswick, NJ, USA).

### 4.2. BV-2 Cell Culture and Treatment

The mouse microglial cell line BV-2 (ATCC, Rockville, MD, USA) was routinely cultured in Roswell Park Memorial Institute (RPMI) 1640 medium, supplemented with 10% fetal bovine serum (FBS), 100 U/mL penicillin, 100 mg/mL streptomycin and 2 mM l-glutamine at 37 °C in a humidified 5%-CO_2_ incubator. In all experiments, the cells were seeded at a density of 1 × 10^5^ cells/mL. After seeding for 24 h, the culture medium was replaced with serum-free RPMI (Roswell Park Memorial Institute) 1640 medium, pretreated with different doses of EGCG (5, 10, 20 μM) for 2 h, and finally incubated with 200 μM PA. After 24 h of PA treatment, the growth medium was collected for detecting the inflammatory cytokines. Cellular proteins were extracted for further western blot analysis.

### 4.3. Oil Red O Staining

BV-2 cells were fixed with 10% formaldehyde in PBS for 10 min and then stained with fresh oil red O solution (Solarbio, Beijing, China) for 10 min at room temperature. After washing with distilled water, the stained cells were inspected under a microscope (Zeiss, Oberkochen, Germany). The droplets were subsequently dissolved in isopropanol, and the lipid content was quantified by measuring the absorbance at 490 nm. ImageJ software (NIH, Bethesda, MD, USA) was applied for analyzing the gray value.

### 4.4. Animals

All animals were handled following the guidelines of the National Institutes of Health (NIH) for animal care and the use of laboratory animals. Also, the animal experiments were approved by the Animal Care and Use Committee at Zhejiang University (ethic approval code: ZJU20190071). Twenty four-week-old male C57BL/6J mice were purchased from Shanghai SLAC Laboratory Animal Co., Ltd. (Shanghai, China). The mice were randomly assigned to four groups (*n* = 6/group) with free access to food and water in standardized conditions (12:12 h of the light-dark cycle, the temperature of 22 ± 2 °C with a relative humidity of 55 ± 5%). After a one-week-adaptation, four groups of mice were fed on the following diets for eight weeks: a normal chow diet (NCD, with 10% of energy from fat), a normal chow diet blending with 1% EGCG (NCD + EGCG, with 10% of energy from fat), a high-fat diet (HFD, with 60% of energy from fat), and a high-fat diet blending with 1% EGCG (HFD + EGCG, with 60% of energy from fat). Th body weights of the mice of four groups were recorded weekly.

### 4.5. Collection of Serum and Tissue Samples

At the end of the eighth week of the experiment, the serum of the mice was collected after a fast of 12 h for determining the serum glucose and lipid levels using an automatic biochemical analyzer (TBA-40FR, Toshiba Medical, Tokyo, Japan). Epididymal adipose tissues and hypothalamus samples were collected, immediately frozen in liquid nitrogen, and stored at −80 °C for further histology, gene expression, ELISA, and western blot studies.

### 4.6. H&E Staining

Epididymal adipose tissues were washed in saline and fixed with 4% paraformaldehyde for 24 h before embedding in paraffin. Embedded adipose tissues were sectioned and stained with hematoxylin and eosin (H&E), and observed under a microscope (Zeiss, Oberkochen, Germany) at a magnification of ×200.

### 4.7. Western Blot Analysis

BV-2 cells and tissues were lysed in RIPA buffer, containing protease and phosphate inhibitors (Thermo Scientific, Rockford, IL, USA). Protein concentrations in the cell and tissue homogenates were determined using a BCA protein assay kit (Thermo Scientific, Rockford, IL, USA). Equal quantities (40 μg) of proteins were separated on a 10% Sodium Dodecyl Sulfate (SDS)-polyacrylamide gel, transferred onto Polyvinylidene Fluoride (PVDF) membranes and blocked with 5% non-fat milk in TBST for 1 h at room temperature. The protein abundance was detected after incubating the primary antibodies against STAT3, phospho-STAT3, JAK2, phospho-JAK2, followed by HRP-conjugated secondary antibody. The blots were visualized by an enhanced chemiluminescence (ECL) detection system (Bio-Rad, Hercules, CA, USA).

### 4.8. Enzyme-Linked Immunosorbent Assay (ELISA)

The levels of TNF-α, IL-6, and IL-1β in culture supernatants and hypothalamic homogenate were measured by ELISA kit (R&D Systems, Minneapolis, MN, USA) according to the manufacturer’s instructional manual, and the OD values were measured at 450 nm. The concentration of each cytokine was calculated from the linear equation derived from the standard curve of the recombinant cytokine in the kit.

### 4.9. Immunofluorescence

Immunofluorescence staining was performed for measuring the expression of Iba-1 in hypothalamic ARC and PVN as described in our previous research [25]. Ithe mageJ software (NIH, Bethesda, MD, USA) was applied for analyzing the cell number, cell size, and fluorescence intensity.

### 4.10. Statistical Analysis

Data are presented as means ± standard error of the mean (SEM). Statistical analyses were carried out with one-way ANOVA followed by Tukey’s post hoc test using SPSS version 18.0 (IBM Corporation., Armonk, NY, USA). For multiple comparisons, statistical differences among the groups are indicated with superscript letters. Differences between the means were considered statistically significant at *p* < 0.05.

## Figures and Tables

**Table 1:** Effects of (−)-epigallocatechin gallate (EGCG) on serum biochemical parameters.

Serum Biochemical Indices	NCD	NCD + EGCG	HFD	HFD + EGCG
Glucose (mmol/L)	5.93 ± 0.57 ^c^	6.02 ± 0.48 ^c^	8.82 ± 0.66 ^a^	6.78 ± 0.34 ^b^
TG (mmol/L)	1.16 ± 0.15 ^b^	1.24 ± 0.11 ^b^	1.38 ± 0.14 ^a^	1.15 ± 0.19 ^b^
TC (mmol/L)	3.10 ± 0.25 ^c^	2.91 ± 0.15 ^c^	4.36 ± 0.43 ^a^	3.44 ± 0.16 ^b^
HDL (mmol/L)	2.96 ± 0.34 ^a^	2.75 ± 0.16 ^a^	2.89 ± 0.29 ^a^	3.14 ± 0.30 ^a^
LDL (mmol/L)	0.31 ± 0.09 ^a^	0.29 ± 0.03 ^a^	0.39 ± 0.11 ^a^	0.24 ± 0.08 ^a^

NCD, a normal chow diet; NCD + EGCG, a normal chow diet supplemented with 1% EGCG; HFD, a 60 kcal% high-fat diet; HFD + EGCG, a 60 kcal% high-fat diet supplemented with 1% EGCG. Data are presented as mean ± SEM (*n* = 6). Superscript letters a, b and c indicate significantly different means at *p* < 0.05 (a > b > c).

## Abbreviations

AgRP	Agouti-related neuropeptide
ARC	Arcuate nucleus
CNS	Central nervous system
DIO	Diet-induced obesity
EGCG	(−)-Epigallocatechin gallate
HFD	High-fat diet
IL-6	Interleukin-6
IL-1β	Interleukin 1-beta
JAK2	Janus kinase 2
PA	Palmitic acid
POMC	Proopiomelanocortin
PVN	Paraventricular nucleus
SFA	Saturated fatty acids
STAT3	Signal transducers and activators of transcription 3
TNF-α	Tumor necrosis factor-alpha
